# The Evidence for Hypoperfusion as a Factor in Multiple Sclerosis Lesion Development

**DOI:** 10.1155/2013/598093

**Published:** 2013-04-04

**Authors:** Bernhard H. J. Juurlink

**Affiliations:** ^1^Department of Anatomy & Cell Biology, University of Saskatchewan, Saskatoon, SK, Canada S7N 5E5; ^2^College of Medicine, Alfaisal University, Riyadh 11533, Saudi Arabia

## Abstract

The evidence that hypoxia is a precipitating factor in causing early MS lesions includes increased protein levels of hypoxia-inducible factor-1**α**; presence of the D-110 hypoxia-inducible protein; increased expression of hypoxia-inducible genes in lesions as well as in adjacent normal-appearing white matter (NAWM); loss of myelin-associated glycoprotein in myelin of early MS lesions; a 50% reduction of blood flow through NAWM with areas of lowest blood flow having the greatest probability of lesion development. Why MS-like lesions develop following hypoxemic insults in some individuals but not in others is likely dependent upon the presence of immune predisposing factors that are governed genetically. Hypoperfusion may be due to decreased arterial supply, restricted venous return, or a combination of these. There are clinical trials ongoing or planned to treat chronic cerebrospinal venous insufficiency (CCSVI) through angioplasty. I suggest that it is important that clinical trials addressing vascular issues in MS should examine how the vascular intervention affects white matter perfusion and determine whether the extent of perfusion recovery and maintenance of this recovery is related to functional recovery and maintenance of functional recovery. Consideration should also be given to the possibility of arterial problems playing a role in hypoperfusion in some MS patients.

## 1. Introduction

Multiple sclerosis (MS) is a complex disease with both environmental and genetic factors playing a role in the disease [1]. Most of the current therapeutic approaches in treating MS are based upon the thinking that the primary disorder is in immune regulation [2]. This, however, does not take into account that the first identifiable feature of MS is a breakdown in the blood-brain barrier (BBB) and that this can also occur in the retina [3], a site that does not contain myelin. Considerable evidence has accumulated over the past few decades suggesting that the immune attack on myelin may be secondary to damage of oligodendrocytes and associated myelin [4, 5]. The question is what causes this oligodendrocyte damage? This paper reviews the evidences that suggest that hypoperfusion might be a causal factor in oligodendrocyte and myelin damage that results in a frank immune attack on these structures in individuals with specific genetic backgrounds. This might explain a possible linkage between chronic cerebrospinal venous insufficiency (CCSVI) and MS [6]. There is considerable controversy whether CCSVI is a predisposing factor for MS; nevertheless, due to patient demand, there are a number of clinical trials underway, or planned, involving venous angioplasty as a treatment for MS. If hypoxia plays a role in promoting MS lesion development, then attention needs to be paid to white matter perfusion as an outcome of treatment and whether improvements in perfusion correlate with improvements in function.

## 2. Lesion Patterns in MS

Lucchinetti and colleagues classified MS lesions into four distinct patterns [7]. Patterns I and II displayed the typical perivenous demyelinated lesions with activated macrophages and lymphocyte involvement. Pattern III was characterized by a distal oligodendrocyte dystrophy with myelin-associated glycoprotein (MAG) loss and oligodendrocyte apoptosis and demyelination not centred on inflamed blood vessels. Pattern IV lesions were present only in primary progressive MS and the lesions resembled those present in pattern I and II. The authors suggested that these patterns might represent four distinct subgroups of MS or represent distinct stages of the disease. This latter interpretation is likely the correct one. Barnett and Prineas have conducted a series of studies on early MS lesions and conclude that pattern III is characteristic of early lesions where one sees early myelin loss with few or no parenchymal T and B cells whereas it is in later lesions where one sees active demyelination, activated macrophages and numerous immune cells [8–10]. A study by Breij et al. concludes that pattern II is characteristic of active demyelinating lesions [11]. The remaining discussion in this paper is based upon interpreting pattern III MS lesions as being early lesions. 

## 3. Evidence for Hypoxia in Early MS Lesions

An indication of CNS damage is the loss of myelin-associated glycoprotein (MAG) from the inner myelin membranes in early lesions and adjacent apparently normal appearing white matter (NAWM) of MS patients [12]. MAG loss is also seen following ischemic insults such as stroke [12] suggesting that ischemia is a causal factor in MAG degradation. MAG loss in early MS lesions and adjacent white matter as well as following stroke is coincident with nuclear presence of hypoxia-inducible factor-1*α* (HIF1*α*) [12]. HIF1*α* protein levels increase with decreasing oxygen tension [13], hence, suggesting that MAG loss may be directly related to hypoxia. A hypoxia-inducible protein recognized by the monoclonal antibody D-110 is also seen in pattern III MS lesions and adjacent NAWM [14, 15]. As HIF1*α* protein levels increase, it partners with HIF1*β* and translocates to the nucleus where the heterodimer binds to hypoxia-inducible promoter elements promoting expression of hypoxia-inducible genes such as vascular endothelial cell factor [16]. Increased levels of transcripts for HIF1-inducible genes such as glucose transporter-3 as well as vascular endothelial growth factor and its receptor-1 are seen in NAWM adjacent to MS lesions [17]. Also congruent with hypoxia is increased blood vessel density and increased endothelial cell proliferation seen in NAWM of MS patients [18].

The redox changes associated with hypoxia can also cause endoplasmic reticulum (ER) stress [19]. The ER stress-associated proteins such as C/EPB homologous protein and glucose-regulated protein-78 (also known as BiP) are seen in early demyelinating lesions as well as in adjacent nonlesion white matter [15, 20]. Finally, hypoxia also increases the permeability of the BBB [21], likely in response to vascular endothelial growth factor, and causes expression of pro-inflammatory genes in endothelium [22], likely through activation of nuclear factor kappa B (NF*κ*B), thereby promoting expression of pro-inflammatory genes [16]. This suggests the possibility that hypoxia may be responsible for BBB alterations seen in MS. ER stress protein expression and alterations in the BBB do not necessarily indicate hypoxia but are fully congruent with the presence of hypoxia. Furthermore, studies in rats have shown that reductions in blood flow to the cerebral hemispheres by 50% results in white matter damage with axon sparing [23]. Oligodendroglia are known to be especially susceptible to ischemic insults [24, 25]. A recent study in mice has demonstrated that even mild chronic hypoperfusion (blood flow reduction of 15%–25%) results in disruption of axon-myelin integrity with maldistribution of MAG as well as upregulation of pro-inflammatory genes [26].

The major deleterious effects of hypoxia are due to an inability to synthesize enough ATP for maintenance of cellular function. Stys and Trapp [27, 28] have proposed that the hypoxia-like damage found in MS lesions is due to a condition of “virtual” hypoxia. The “virtual” hypoxia is an energy deficit caused by increased energy demand coupled with impaired mitochondrial ability to synthesize ATP. Thus, one can have “virtual” hypoxia in the presence of oxygen. Although a condition of “virtual” hypoxia may contribute to damage, particularly axonal damage, in an MS lesion, it cannot account for increased HIF1*α* protein levels and associated increase in expression of HIF-inducible genes. HIF1*α* protein is constantly being synthesized and under normal oxygen tension two proline residues within the oxygen-dependent degradation domain are hydroxylated by prolyl hydroxylases [13]. The hydroxylated proline residues interact with the von Hippel-Lindau tumour suppressor protein allowing polyubiquination and subsequent degradation via the 26S proteasome complex. As oxygen levels become lower, the hydroxylation of proline residues becomes less efficient and increases the half-life of HIF1*α* protein enabling it to heterodimerize with HIF1*β* protein, thus allowing translocation of the heterodimer to the nucleus where it binds to hypoxia-inducible elements. Thus, “virtual” hypoxia cannot account for the increased HIF1*α* protein levels seen in and around MS lesions, nor the D-110 antigen.

All the previous evidence, despite being indirect, is congruent with the hypothesis that hypoxia, and not just “virtual” hypoxia, plays a causal role in early MS lesion formation. Is there direct evidence? Many studies, using magnetic resonance imaging approaches, have found an impairment of about 50%, or more, of blood flow through NAWM of MS patients [29–34]. Furthermore, the probability of developing MS lesions is greater in NAWM with the lowest perfusion rates [34]. One could argue that in MS, large areas of white matter may not function normally, thereby accounting for the reduced blood flow. Indeed, reviews on vascular aspects of MS by De Keyser and colleagues [35] and D'haeseleer and colleagues [36] present evidence of dysfunction in astrocytes that could account for reduced blood flow, thus accounting for hypoperfusion. Astrocytes play important roles in regulating cerebral blood flow [37], and it is possible that dysfunction of astrocytes could result in hypoperfusion severe enough to result in hypoxia. Saindane and colleagues in an MRI study of remitting-relapsing MS patients and control subjects conclude, based on the correlation between decreased white matter perfusion and decreased mean diffusivity with no changes in fractional anisotropy (indicative of no structural changes), that reduced NAWM blood flow is a primary event and not due to decreased tissue metabolism [38]. It is possible, however, that functional impairments of astrocyte-mediated regulation of blood flow need not necessarily give rise to structural changes that can be detected using MRI. 

## 4. Possible Mechanisms Involved in Initiation of MS Lesion Development

There is now an abundance of evidence that hypoxia and hypoperfusion are associated with early lesions and NAWM of MS patients. The major question is whether this is part of the consequences of MS lesion development or a causal factor in MS lesion development. One can easily envision that lesion development is preceded by impairment of blood flow through white matter. This impairment may be due to restricted venous return, impaired arterial supply, or a combination of these factors. When flow is impaired sufficiently, it results in (1) increased levels of HIF1*α* and activation of NF*κ*B expression leading to expression of hypoxia-inducible genes and pro-inflammatory genes in endothelium and neural cells; (2) breakdown of the blood-brain barrier mediated in part by the hypoxia-inducible vascular endothelial cell growth factor; (3) activation of microglia and astrocytes; (4) damage to oligodendrocytes, and so forth, as evidenced by loss of MAG and subsequent apoptosis. These changes would occur in the absence of significant leukocyte involvement as evidenced in pattern III lesions. Pro-inflammatory chemokines and cytokines expression by endothelium and glial cells activate and attract immune cells to the site of the developing lesion, while cell adhesion molecules expressed on postcapillary venules allow the leukocytes to infiltrate the incipient lesion where they encounter myelin breakdown products resulting in frank immune attack. One possible concern with this schema is that hypoperfusion and subsequent oligodendrocyte/myelin damage and expression of pro-inflammatory genes in the sites affected following a stroke do not result in an MS-like immune attack on myelin. Clearly, there is an immune predisposition to developing MS as it is indicated that the majority of the gene variants that increase the probability of developing MS are immune-related [39, 40]. 

## 5. Clinical Trials to Treat Chronic Cerebrospinal Venous Insufficiency (CCSVI)

Zamboni and colleagues reported, using transcranial Doppler sonography, that a significant proportion of remitting-relapsing and progressive MS patients had reduced venous return in the deep middle cerebral vein with evidence of reflux [6]. Subsequently, where Doppler studies were combined with selective venography and the control group also included non-MS neurological patients, it was reported that 71% of MS patients had reflux in either the internal jugular or vertebral veins, and 61% had reflux in one of the deep cerebral veins, with 86% of the patients having a stenosis somewhere along the azygous vein and 37% of the patients having a stenosis of one or both proximal internal jugular veins [41]. These conditions were labeled as CCSVI. This was followed by a manuscript examining the safety of angioplasty on the constricted veins in MS patients and clinical improvements following angioplasty [42]. CCSVI should increase postcapillary venous pressure and decrease perfusion across the capillary bed resulting in hypoxemia. 

A large number of studies on CCSVI have been now reported from the Zamboni clinic as well as other clinics. Many of these studies could not demonstrate an increased incidence of CCSVI in MS, for example, the review by Baracchini and colleagues [43], whereas other studies find a greatly increased incidence of CCSVI in MS patients, for example, [44]. The differences in findings amongst different studies may be due to differing abilities to detect CCSVI.

 Whether CCSVI plays a role in MS or not is still controversial; however, there are a number of clinical trials to test whether angioplasty in MS patients with CCSVI is underway or is about to be underway—see NIH Clinical Trials Registry. If these trials are not properly conducted, then all the information that could be of value to understanding MS may not be collected. What will be critical in such trials is the ability to accurately diagnose CCSVI. Doppler studies are very operator dependent and possibly account for the great differences in CCSVI incidence seen amongst the various studies. Perhaps the MRI protocol used in the recent study by Haacke and colleagues [45] that included a coronal 3D time-resolved contrast enhanced MR arteriovenography followed by transverse time-of-flight venography and 2D phase contrast MR scan for flow quantification should be used for diagnosis of CCSVI.

Anecdotal evidence from patients suggests that angioplasty improves functional outcome in only a subset of patients and may worsen symptoms in a very small proportion of patients. This observation is supported by a nonrandomized, nonblinded, and non-placebo-controlled angioplasty study of MS patients where at 1 month following treatment 68% had improvements using the Multiple Sclerosis Impact Scale-29 (MSIS-29) physical score while 6% were worse off after angioplasty [46]. If carefully controlled blinded, placebo-controlled clinical trials indicate that there is significant functional improvement in patients, or a subset of patients, then it becomes very important to determine whether the extent of improved functional outcome correlates with improved perfusion. If clinical trials demonstrate that the extent of functional recovery following angioplasty in CCSVI patients is directly related to recovery of white matter perfusion, then thoughts should be directed towards promoting perfusion in MS patients who do not exhibit the characteristics of CCSVI where the perfusion problem may be related to arterial disturbances. 

## 6. Concluding Remarks

The dominant perspective on MS in recent decades is that MS is an autoimmune disease with the primary problem being an immune dysfunction. This has very much been influenced by experimental allergenic encephalomyelitis animal models. Treatments for MS are mainly directed towards immune modulation [47]. Interferon beta-1 immunotherapy is one of the oldest immune-modulating treatments. Although administration of interferon beta to MS patients results in fewer MRI-detectable lesions and fewer relapses, treatment with interferon beta-1 has no effect on disability progression, neither in remitting-relapsing MS [48], nor in secondary progressive MS [49]. Despite this, many researchers and clinicians have difficulty to consider the possibility that the immune response seen in MS may be secondary to some change in the CNS.

Until Dr. Zamboni published his ideas on the relationship between CCSVI and MS, most MS investigators of the past few decades, as noted earlier, focused on MS as an autoimmune disorder and had little knowledge of the historical focus of MS research on the involvement of the vasculature, particularly veins, in lesion development see review by Haacke [50]. That venous obstruction may play a role in MS lesion was first proposed by Putnam who demonstrated that venular obstruction in dogs led to MS-like lesion formation [51]. Thus, a primary venular involvement in MS lesion development is not a new idea.

As outlined in this paper, there is an abundance of evidence that hypoxia is associated with pattern III lesions. Whether hypoxia is a consequence of the disease process or plays a causal role is yet to be determined, but it is conceivable that hypoxia may play a causal role. A reasonable interpretation of pattern III lesions is that they are early lesions. Hypoxia can account for BBB breakdown and initial oligodendrocyte/myelin damage. NAWM of MS patients experiences greatly lowered blood flow and, thus, must experience a degree of hypoxia as is also suggested by increased expression of hypoxia-inducible genes. That pattern III lesions are not perivenous while well-established lesions are perivenous is likely explained by the fact that immune cells migrate from the blood to the parenchyma via postcapillary venules where they then, encountering damaged myelin, mount an immune attack resulting in the establishment frank demyelinating perivenous lesions. The evidence is sufficient to seriously consider the possibility that hypoperfusion is a precipitating factor in MS lesions and to examine whether improvements in white matter perfusion correlates with improvements of functional outcomes.

## References

[1] Taylor BV (2011). The major cause of multiple sclerosis is environmental: genetics has a minor role—yes. *Multiple Sclerosis*.

[2] Gonzalez-Andrade F, Alcaraz-Alvarez JL (2010). Disease-modifying therapies in relapsing-remitting multiple sclerosis. *Neuropsychiatric Disease and Treatment*.

[3] Birch MK, Barbosa S, Blumhardt LD, O’Brien C, Harding SP (1996). Retinal venous sheathing and the blood-retinal barrier in multiple sclerosis. *Archives of Ophthalmology*.

[4] Juurlink BHJ (1998). The multiple sclerosis lesion: initiated by a localized hypoperfusion in a central nervous system where mechanisms allowing leukocyte infiltration are readily upregulated?. *Medical Hypotheses*.

[5] Moore GR (2010). Current concepts in the neuropathology and pathogenesis of multiple sclerosis. *The Canadian Journal of Neurological Sciences*.

[6] Zamboni P, Menegatti E, Bartolomei I (2007). Intracranial venous haemodynamics in multiple sclerosis. *Current Neurovascular Research*.

[7] Lucchinetti C, Bruck W, Parisi J, Scheithauer B, Rodriguez M, Lassmann H (2000). Heterogeneity of multiple sclerosis lesions: implications for the pathogenesis of demyelination. *Annals of Neurology*.

[8] Barnett MH, Prineas JW (2004). Relapsing and remitting multiple sclerosis: pathology of the newly forming lesion. *Annals of Neurology*.

[9] Henderson APD, Barnett MH, Parratt JDE, Prineas JW (2009). Multiple sclerosis: distribution of inflammatory cells in newly forming lesions. *Annals of Neurology*.

[10] Prineas JW, Parratt JD (2012). Oligodendrocytes and the early multiple sclerosis lesion. *Annals of Neurology*.

[11] Breij ECW, Brink BP, Veerhuis R (2008). Homogeneity of active demyelinating lesions in established multiple sclerosis. *Annals of Neurology*.

[12] Aboul-Enein F, Rauschka H, Kornek B (2003). Preferential loss of myelin-associated glycoprotein reflects hypoxia-like white matter damage in stroke and inflammatory brain diseases. *Neuropathology and Experimental Neurology*.

[13] Greer SN, Metcalf JL, Wang Y, Ohh M (2012). The updated biology of hypoxia-inducible factor. *EMBO Journal*.

[14] Lassmann H, Reindl M, Rauschka H (2003). A new paraclinical CSF marker for hypoxia-like tissue damage in multiple sclerosis lesions. *Brain*.

[15] Mháille AN, McQuaid S, Windebank A (2008). Increased expression of endoplasmic reticulum stress-related signaling pathway molecules in multiple sclerosis lesions. *Neuropathology and Experimental Neurology*.

[16] Taylor CT (2008). Interdependent roles for hypoxia inducible factor and nuclear factor-*κ*B in hypoxic inflammation. *Journal of Physiology*.

[17] Graumann U, Reynolds R, Steck AJ, Schaeren-Wiemers N (2003). Molecular changes in normal appearing white matter in multiple sclerosis are characteristic of neuroprotective mechanisms against hypoxic insult. *Brain Pathology*.

[18] Holley JE, Newcombe J, Whatmore JL, Gutowski NJ (2010). Increased blood vessel density and endothelial cell proliferation in multiple sclerosis cerebral white matter. *Neuroscience Letters*.

[19] Bánhegyi G, Mandl J, Csala M (2008). Redox-based endoplasmic reticulum dysfunction in neurological diseases. *Journal of Neurochemistry*.

[20] Cunnea P, Mháille AN, McQuaid S, Farrell M, McMahon J, Fitzgerald U (2011). Expression profiles of endoplasmic reticulum stress-related molecules in demyelinating lesions and multiple sclerosis. *Multiple Sclerosis*.

[21] Dux E, Temesvari P, Joo F (1984). The blood-brain barrier in hypoxia: ultrastructural aspects and adenylate cyclase activity of brain capillaries. *Neuroscience*.

[22] Schmedtje JF, Ji YS, Liu WL, DuBois RN, Runge MS (1997). Hypoxia induces cyclooxygenase-2 via the NF-*κ*B p65 transcription factor in human vascular endothelial cells. *Journal of Biological Chemistry*.

[23] Farkas E, Donka G, de Vos RAI, Mihály A, Bari F, Luiten PGM (2004). Experimental cerebral hypoperfusion induces white matter injury and microglial activation in the rat brain. *Acta Neuropathologica*.

[24] Husain J, Juurlink BHJ (1995). Oligodendroglial precursor cell susceptibility to hypoxia is related to poor ability to cope with reactive oxygen species. *Brain Research*.

[25] Jelinski SE, Yager JY, Juurlink BHJ (1999). Preferential injury of oligodendroblasts by a short hypoxic-ischemic insult. *Brain Research*.

[26] Reimer MM, McQueen J, Searcy L (2011). Rapid disruption of axon-glial integrity in response to mild cerebral hypoperfusion. *Journal of Neuroscience*.

[27] Stys PK (2004). Axonal degeneration in multiple sclerosis: is it time for neuroprotective strategies?. *Annals of Neurology*.

[28] Trapp BD, Stys PK (2009). Virtual hypoxia and chronic necrosis of demyelinated axons in multiple sclerosis. *The Lancet Neurology*.

[29] Law M, Saindane AM, Ge Y (2004). Microvascular abnormality in relapsing-remitting multiple sclerosis: perfusion MR imaging findings in normal-appearing white matter. *Radiology*.

[30] Varga AW, Johnson G, Babb JS, Herbert J, Grossman RI, Inglese M (2009). White matter hemodynamic abnormalities precede sub-cortical gray matter changes in multiple sclerosis. *Journal of the Neurological Sciences*.

[31] Ge Y, Law M, Johnson G (2005). Dynamic susceptibility contrast perfusion MR imaging of multiple sclerosis lesions: characterizing hemodynamic impairment and inflammatory activity. *American Journal of Neuroradiology*.

[32] Adhya S, Johnson G, Herbert J (2006). Pattern of hemodynamic impairment in multiple sclerosis: dynamic susceptibility contrast perfusion MR imaging at 3.0T. *NeuroImage*.

[33] Inglese M, Adhya S, Johnson G (2008). Perfusion magnetic resonance imaging correlates of neuropsychological impairment in multiple sclerosis. *Journal of Cerebral Blood Flow and Metabolism*.

[34] Holland CM, Charil A, Csapo I (2012). The relationship between normal cerebral perfusion patterns and white matter lesion distribution in 1,249 patients with multiple sclerosis. *Journal of Neuroimaging*.

[35] De Keyser J, Steen C, Mostert JP, Koch MW (2008). Hypoperfusion of the cerebral white matter in multiple sclerosis: possible mechanisms and pathophysiological significance. *Journal of Cerebral Blood Flow and Metabolism*.

[36] D’haeseleer M, Cambron M, Vanopdenbosch L, De Keyser J (2011). Vascular aspects of multiple sclerosis. *The Lancet Neurology*.

[37] Gordon GRJ, Mulligan SJ, MacVicar BA (2007). Astrocyte control of the cerebrovasculature. *GLIA*.

[38] Saindane AM, Law M, Ge Y, Johnson G, Babb JS, Grossman RI (2007). Correlation of diffusion tensor and dynamic perfusion MR imaging metrics in normal-appearing corpus callosum: support for primary hypoperfusion in multiple heterosis. *American Journal of Neuroradiology*.

[39] Sawcer S, Hellenthal G, Pirinen M (2011). Genetic risk and a primary role for cell-mediated immune mechanisms in multiple sclerosis. *Nature*.

[40] Wang JH, Pappas D, De Jager PL (2011). Modeling the cumulative genetic risk for multiple sclerosis from genome-wide association data. *Genome Medicine*.

[41] Zamboni P, Galeotti R, Menegatti E (2009). Chronic cerebrospinal venous insufficiency in patients with multiple sclerosis. *Journal of Neurology, Neurosurgery and Psychiatry*.

[42] Zamboni P, Galeotti R, Menegatti E (2009). A prospective open-label study of endovascular treatment of chronic cerebrospinal venous insufficiency. *Journal of Vascular Surgery*.

[43] Baracchini C, Atzori M, Gallo P (2012). CCSVI and MS: no meaning, no fact. *Neurological Sciences*.

[44] Utriainen D, Feng W, Elias S, Latif Z, Hubbard D, Haacke EM (2012). Using magnetic resonance imaging as a means to study chronic cerebral spinal venous insufficiency in multiple sclerosis patients. *Techniques in Vascular and Interventional Radiology*.

[45] Haacke EM, Feng W, Utrianen D (2012). Patients with multiple sclerosis with structural venous abnormalities on MRI imaging exhibit an abnormal flow distribution of the internal jugular veins. *Journal of Vascular and Interventional Radiology*.

[46] Hubbard D, Ponec D, Gooding J, Saxon R, Sauder H, Haacke M (2012). Clinical improvement after extracranial venoplasty in multiple sclerosis. *Journal of Vascular and Interventional Radiology*.

[47] Farooqi N, Gran B, Constantinescu CS (2010). Are current disease-modifying therapeutics in multiple sclerosis justified on the basis of studies in experimental autoimmune encephalomyelitis?. *Journal of Neurochemistry*.

[48] Shirani A, Zhao Y, Karim ME (2012). Association between use of interferon beta and progression of disability in patients with relapsing-remitting multiple sclerosis. *Journal of the American Medical Association*.

[49] La Mantia L, Vacchi L, Rovaris M (2013). Interferon beta for secondary progressive multiple sclerosis: a systematic review. *Journal of Neurology, Neurosurgery & Psychiatry*.

[50] Haacke EM (2011). Chronic cerebral spinal venous insufficiency in multiple sclerosis. *Expert Review of Neurotherapeutics*.

[51] Putnam TJ (1935). Studies in multiple sclerosis: encephalitis and sclerotic plaques produced by venular obstructions. *Archives of Neurology & Psychiatry*.

